# Effects of *Pseudomonas aeruginosa* and *Streptococcus mitis* mixed infection on TLR4-mediated immune response in acute pneumonia mouse model

**DOI:** 10.1186/s12866-017-0999-1

**Published:** 2017-04-04

**Authors:** Chao Song, Hongdong Li, Yunhui Zhang, Jialin Yu

**Affiliations:** 1 Department of Neonatology, Children’s Hospital of Chongqing Medical University, Chongqing, China; 2 Ministry of Education Key Laboratory of Child Development and Disorders – Chongqing Key Laboratory of Pediatrics, China International Science and Technology Cooperation Base of Child Development and Critical Disorders, Chongqing, China

**Keywords:** Acute lung infection, *Pseudomonas Aeruginosa*, *Streptococcus Mitis*, TLR4, Il-6, Tnf-α

## Abstract

**Background:**

Our previous research on the diversity of microbiota in the endotracheal tubes (ETTs) of neonates in the neonatal intensive care unit found that *Pseudomonas aeruginosa* (*P. aeruginosa*) and *Streptococcus mitis* (*S. mitis*) were the dominant bacteria on the ETT surface and the existence of *S. mitis* could promote biofilm formation and pathogenicity of *P. aeruginosa*. Toll-like receptor 4 (TLR4), which has been widely detected on the surface of airway epithelial cells, is the important component of the innate immune system. Therefore, we hypothesized that the co-existence of these two bacteria might impact the host immune system through TLR4 signaling.

**Results:**

*S. mitis* rarely caused inflammation, whereas *P. aeruginosa* caused the most severe inflammation accompanied by increases in the number of inflammatory cells, interleukin (IL)-6 and tumor necrosis factor (TNF)-α expression, and total cell counts in BALF (*p* < 0.05). In the PAO1 + *S. mitis* group, moderate inflammation, reduced IL-6 and TNF-α protein levels, and decreased total cell counts were observed. Additionally, levels of these indicators were decreased lower in TLR4-deficient mice than in wild-type mice (*p* < 0.05).

**Conclusions:**

Our results demonstrated that infection with *S. mitis* together with *P. aeruginosa* could alleviate lung inflammation in acute lung infection mouse models possibly via the TLR4 signaling pathway.

## Background

Ventilator-associated pneumonia (VAP) is a common device-related infection in neonatal intensive care units (NICUs), and it is associated with high morbidity and mortality as well as increased antibiotic resistance and high economic costs [1, 2]. Our previous research demonstrated that *Pseudomonas aeruginosa (P. aeruginosa)* and *Streptococcus mitis (S. mitis)* were the most dominant microbes on the surface of neonatal endotracheal tubes (ETTs) [3, 4]. In addition, the co-existence of *S. mitis* from the oral microbiome and the opportunistic pathogen *P. aeruginosa* on the same ETT may play a crucial role in biofilm formation. Khosravi et al. found that *S. mitis* produced and released a tenovin 6-like molecule that induced growth inhibition and coccoid conversion of *Helicobacter pylori* cells [5]. Moreover, recent discoveries regarding the pathogenesis of VAP has found an interesting phenomenon in which autoinducer-2 produced by *S. mitis* can act as an important molecule to promote *P. aeruginosa* biofilm formation and to increase proinflammatory cytokine secretion in endotracheal intubation rat models [6]. Given that *S. mitis* is the most abundant bacteria of the normal human oral flora and rarely causes diseases, we questioned whether *S. mitis* could also modulate the pathogenicity of *P. aeruginosa* in acute lung infection. If so, how does this co-existence impact the immune system of the host and what is the possible mechanism in acute lung infection?

Toll-like receptors (TLRs), one of the most important receptor families of the innate immune system, can recognize pathogen-associated molecular patterns (PAMPs), including microbial products, and play an important role in the immune response [7, 8]. At present, 11 TLRs have been identified in humans [9]. Recent studies have confirmed that TLR2, 4, 5, and 9 play pivotal roles in the response to bacterial infections [10]. TLR4 is a receptor for lipopolysaccharide (LPS), an important cell wall component of gram-negative bacteria. Through ligand binding, TLR4 recruits signaling adaptors and initiates signaling cascades, which results in the activation of nuclear factor (NF)-κB and the release of inflammatory cytokines, such as interleukin (IL)-6 [11] and tumor necrosis factor (TNF)-α [12]. Moreover, airway epithelial cells are believed to contribute to the inflammatory response in the lung, and TLR4 has been widely detected on the surface of airway epithelial cells as well as cells of the myeloid lineage, such as macrophages and neutrophils [13, 14]. Some studies have reported that TLR4-deficient mice show increased lung inflammation and higher bacterial load, and TLR4 signaling may have a critical function in the fine tuning of inflammation during chronic mycobacterial infection [15]. Therefore, we hypothesized that TLR4 signaling might participate in the response to acute lung infection.

Based on our previous findings that *P. aeruginosa* and *S. mitis* are the dominant bacteria in the mixture of organisms causing lung infections, these two bacteria were selected for the present study. The aim of our study was to explore the relationship between *P. aeruginosa* and *S. mitis* in lung infection and ascertain their roles in the immune response. After acute lung infection mouse models were established, lung bacteriological and histopathological examinations were performed, and total cell counts and levels of related cytokines in bronchoalveolar lavage fluid (BALF) were determined.

## Methods

### Bacteria and growth conditions

*P. aeruginosa* strain PAO1 (ATCC27853) and *S. mitis* (ATCC49456) were used in our study. PAO1 was kindly provided by Professor Li Shen (Institute of Molecular Cell and Biology, New Orleans, LA, USA). *S. mitis* was purchased from American Type Culture Collection. The bacterial strains were both cultured overnight in brain-heart infusion (BHI) broth. The *S. mitis* strain was grown at a neutral pH at 37 °C in a 5% CO_2_ atmosphere. The PAO1 strain was grown at 37 °C on an orbital shaker at 200 rpm. Overnight-grown cultures of the strains were standardized to 0.2 (OD_600_), and then diluted to a working concentration of OD_600_ = 0.1, if necessary.

### Animals

Forty C57BL/6 mice (6–8 weeks, 18–20 g) were obtained from the Experimental Animal Center of Chongqing Medical University and housed in the Laboratory Animal Center at the Children’s Hospital of Chongqing Medical University. Twenty TLR4-deficient mice (C57BL/10ScNJ) were obtained from the Key Laboratory of Diagnostic Medicine Designated by the Ministry of Education at Chongqing Medical University. TLR4 gene expression was determined by polymerase chain reaction (PCR) before the experiments to confirm the reliability of the TLR4-deficient mice. The experimental protocol was approved by the Animal Care and Use Committee of Chongqing Medical University.

### Acute lung infection mouse models

Twenty wild-type mice were randomly divided into four groups (*n* = 5/group) treated with: PAO1 (OD_600_ = 0.1, 1 × 10^8^ CFU/mL, 50 μL), *S. mitis* (OD_600_ = 0.1, 1 × 10^8^ CFU/mL, 50 μL), PAO1 + *S. mitis* (OD_600_ = 0.2, 2 × 10^8^ CFU/mL, 25 μL + 25 μL), or phosphate-buffered saline (PBS) (control group, 50 μL). Twenty TLR4-deficient mice were divided into the same treatment groups. Additionally, another 20 wild-type mice were subjected to the same treatments and used to estimate the bacterial burden, in order to confirm successful establishment of the acute lung infection mouse models.

The mouse models of acute lung infection were established as previously described with some modifications [16]. Briefly, mice were anesthetized with an intraperitoneal injection of chloral hydrate (0.02 mL/g) before tracheotomy. Then they were infected with an intratracheal instillation of 50 μL of bacterial suspension and kept upright in standing posture for 10 s to ensure the bacteria were fully delivered to the lung tissues. Finally, the trachea and skin were sutured. The mice were kept for 48 h before sacrificed.

### Lung bacteriological and histological examinations

The whole lung of each mouse (*n* = 5 mice/group) was homogenized in 1 mL PBS and then serially diluted. A 50-μL sample from each tissue homogenate specimen was cultured quantitatively on Columbia sheep blood agar plates overnight at 37 °C in a 5% CO_2_ atmosphere, and then the colonies were counted to estimate the number of colony-forming units (CFU). The right lungs of the other five mice in each group were fixed in 10% formalin buffer for 48 h, embedded in paraffin, dehydrated, cut into 5-μm slices, and stained with hematoxylin-eosin (H&E) for histopathological examination [17]. Images were obtained using light microscopy (Nikon eclipse 55i, Japan).

### Total cell counts and cytokine analyses in BALF

The left lungs were lavaged and collected with 1 mL PBS. The fluid was instilled and withdrawn three times, and then the total cell counts in the BALF were determined using a cell counter (Countstar, Beijing, China). BALF samples were centrifuged at 3000 g and 4 °C for 5 min. The concentrations of pro-inflammatory cytokines TNF-α and IL-6 in the BALF supernatant were determined using mouse cytokine enzyme-linked immunosorbent assay (ELISA) kits (Beijing 4A Biotech Co., Ltd., Beijing, China) according to the manufacturer’s instructions.

### Statistical analysis

Statistical analyses were carried out using SPSS 22.0 (SPSS, Inc., Chicago, IL, USA). Analysis of variance (ANOVA) was used to identify significant differences among all groups. *p* < 0.05 was considered statistically significant.

## Results

### Bacterial CFU in lung tissues

To confirm the successful establishment of the acute lung infection mouse models, the bacterial burdens in harvested lung tissues were estimated. As shown in Table 1, the numbers of CFU in the PAO1 and PAO1 + *S.mitis* groups were significantly higher than that in the PBS control group (*p* = 0.01 and *p* = 0.01, respectively). More interestingly, although more bacteria were injected initially in the PAO1 + *S. mitis* group, after 48 h, the numbers of CFU did not differ significantly between the PAO1 and PAO1 + *S. mitis* groups (*p* > 0.05). Additionally, the number of CFU in the *S. mitis* group did not differ from that in the PBS control group (*p* > 0.05).

**Table 1:** Bacterial counts in lung tissues of wild-type mice

	PBS	PAO1	*S. mitis*	PAO1 + *S. mitis*
CFU	0 (0–0)	1000 (500–3466)●▲	0 (0–173)	200 (166–800)●▲

●: Significantly different compared to the PBS control group *(p* < 0.05)

▲: Significantly different compared to the *S. mitis* group (*p* < 0.05)

### Histological observation of lung tissues from acute lung infection mouse models

**Fig. 1:**
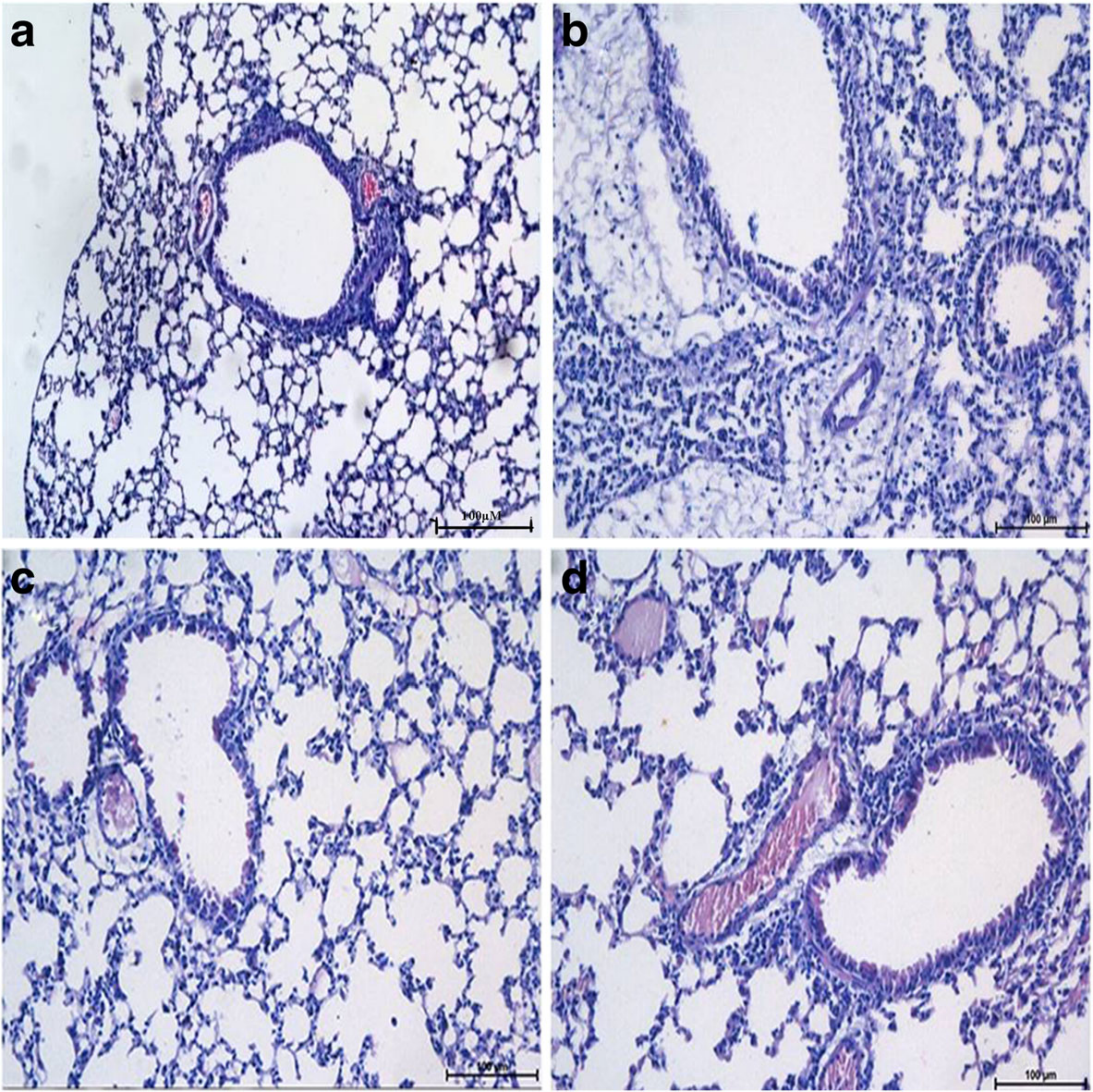
HE-stained lung tissues from wild-type acute lung infection mouse models. After the wild-type mice were infected with PAO1 (**b**), *S. mitis* (**c**), PAO1 + *S. mitis* (**d**), or PBS (**a**) for 48 h, mice were sacrificed and right lungs were stained with HE to observe histological changes. Original magnification, ×200

**Fig. 2:**
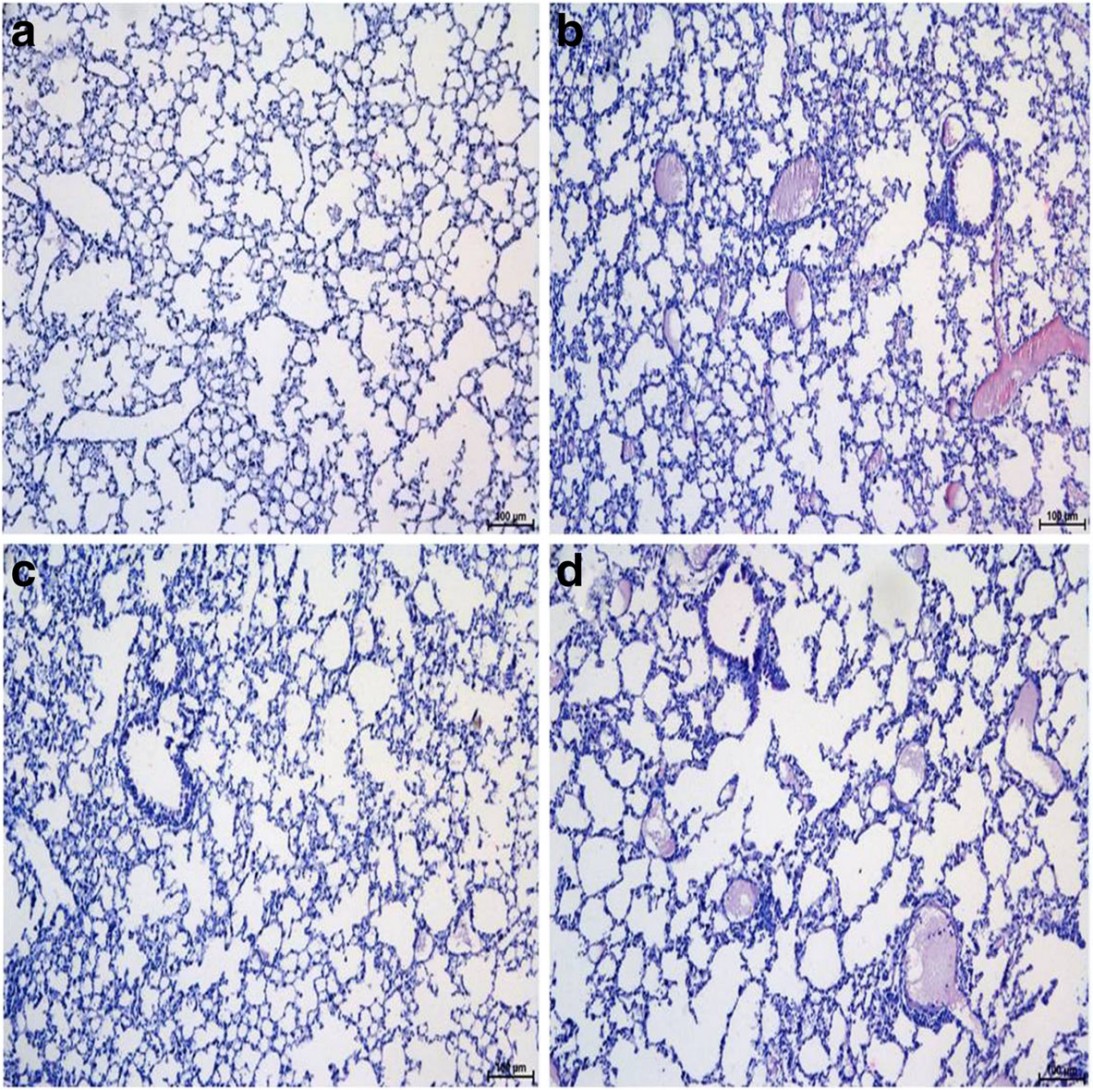
HE-stained lung tissues from TLR4-deficient acute lung infection mouse models. After the TLR4-deficient mice were infected with PAO1 (**b**), *S. mitis* (**c**), PAO1 + *S. mitis* (**d**), or PBS (**a**) for 48 h, mice were sacrificed and right lungs were stained with HE to observe histological changes. Original magnification, ×100

Wild-type mice had severe lung damage after *P. aeruginosa* challenge (Fig. 1b). Numerous foci of necrosis and inflammatory infiltrates were discovered, with increased numbers of alveolar macrophages infiltrating the alveolar septa, as compared with the PBS group. However, little change was observed upon infection of wild-type mice with *S. mitis*, with almost no macrophage infiltration in the lungs (Fig. 1c). Notably, in the PAO1 + *S. mitis* group, moderate lung inflammation was observed, with recruitment of inflammatory cells in the peribronchial wall and surrounding the vessels (Fig. 1d). Similar to wild-type mice, TLR4-deficient mice infected with *S. mitis*, PAO1 + *S. mitis*, or PAO1 showed slight, moderate, and severe lung inflammation, respectively (Fig. 2).

### Cell counts in BALF

**Fig. 3:**
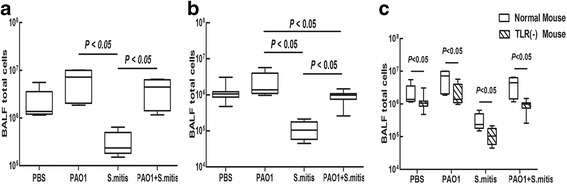
Total cell counts in BALF. The total cell counts in BALF of wild-type mice (**a**) and TLR4-deficient mice (**b**) and the differences in total cell counts between wild-type and TLR4-deficient mice (**c**) are shown

Among infected wild-type mice, the *S. mitis* group showed little change in the total cell count in BALF, as compared to the PBS group (2.17 ± 1.90(×10^6^) vs 3.2 ± 1.9(×10^5^), *p* > 0.05). However, the total cell counts were slightly increased in the PAO1 and PAO1 + *S. mitis* groups (Fig. 3a). Similar results were obtained in TLR4-deficient mice (Fig. 3b). Interestingly, TLR4-deficient mice infected with PAO1 + *S. mitis* had significantly lower total cell counts in BALF compared with TLR4-deficient mice infected with PAO1 only (*p* < 0.001), which is consistent with the results of histological examination of lung tissues. In comparing wild-type and TLR4-deficient mice (Fig. 3c), we found that the BALF of TLR4-deficient mice showed significantly lower total cell counts in each comparative group (all *p* < 0.05).

### IL-6 and TNF-α protein expression

**Fig. 4:**
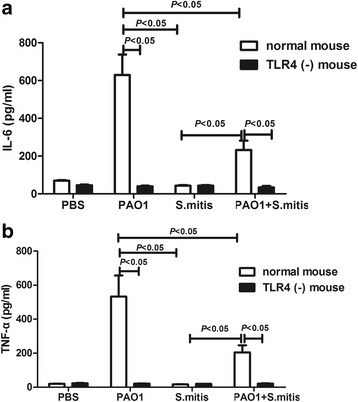
IL-6 and TNF-α concentrations in BALF. The IL-6 protein levels in BALF of wild-type mice and TLR4-deficient mice and the differences in IL-6 protein levels between wild-type and TLR4-deficient mice (**a**) are shown. The TNF-α protein levels in BALF of wild-type mice and TLR4-deficient mice and the differences between in TNF-α protein levels wild-type and TLR4-deficient mice (**b**) are shown

TLR4 recognizes specific bacterial molecules, and their binding initiates host immune responses such as expression of proinflammatory cytokines TNF-α and IL-6. To better understand the immune response in our mouse models, the concentrations of TNF-α and IL-6 were measured. Significantly increased protein levels of IL-6 and TNF-α (*p* < 0.001 and *p* < 0.001, respectively) were observed in wild-type mice challenged with PAO1 compared with levels in the PBS group (Fig. 4). By contrast, *S. mitis* infection hardly induced the secretion of these two cytokines (*p* > 0.05). While increased IL-6 and TNF-α expression was observed the PAO1 + *S. mitis* group relative to the PBS group, these cytokine levels were still lower than those in the PAO1 group (*p* = 0.001 and *p* = 0.002, respectively). Interestingly, after infection of TLR4-deficient with the different bacteria (Fig. 4), expression of both IL-6 and TNF-α was almost completely inhibited, with no statistically significant differences in expression levels among the four treatment groups (*p* > 0.05). In comparison to IL-6 and TNF-α expression levels in wild-type mice, the concentrations of IL-6 and TNF-α were lower in TLR4-deficient mice infected with PAO1 with or without *S. mitis* (*p* < 0.001 and *p* = 0.005, respectively; Fig. 4).

## Discussion

As is known, VAP is a principal cause of morbidity, mortality, and economic burden in ICUs. Increasing antimicrobial resistance has drawn attention to the failure of antibiotic treatment. Recent progress has mainly focused on the pathogenicity and antimicrobial resistance of a single strain, specifically *P. aeruginosa*, on the ETTs. However, few studies have investigated the interaction of multiple bacteria in the pathogenesis of VAP. Based on our previous research, we further investigated the presence of *S. mitis* and *P. aeruginosa* on the ETTs*.* Here, for the first time to our knowledge, we found that *S. mitis* can counteract the inflammatory potential of *P. aeruginosa* possibly through TLR4 signaling in acute lung infection.

*S. mitis* is the most abundant bacteria of the normal human oral flora and also a predominant colonizer of the mucosal site, usually inhabiting the human oral cavity as early as 1–3 days after birth. Moreover, except for endocarditis [18], *S. mitis* rarely causes diseases. In our previous research, we found that wild-type mice as well as TLR4-deficient mice infected with an *S. mitis* strain for 48 h showed little change in pulmonary lesions, supporting the common notion that *S. mitis* is a normal commensal bacteria in the human oropharynx [19–21].

In the present study, we observed more severe inflammation in lung tissues accompanied with infiltration of more inflammatory cells in the PAO1 + *S. mitis* group compared with the *S. mitis* group and PBS control group, but less severe lung inflammation in comparison with that in the PAO1 group. These findings suggest that in acute lung infection, *S. mitis* helps to alleviate the inflammatory response so as to reduce the local tissue damage. Several studies have found similar effects, in which the bacteria mainly act as “protectors” during the process of infection. Recent findings in *Bifidobacteria* and *Lactobacilli* have confirmed that they are effective at reducing allergic symptoms and inhibiting the allergic airway response in murine models of acute airway inflammation [22, 23]. More important, this inhibition is possibly achieved by regulating TLR signaling [24]. Therefore, we have reason to believe that, unlike the traditional concept in which *S. mitis* is considered normal commensal bacteria, our results indicate that *S. mitis* might play a potential protective role in the respiratory tract during acute lung infection. Also considering our results in TLR4-deficient mice, we conclude that *S. mitis* may help alleviate lung inflammation, possibly through modulation of TLR pathway signaling.

Additionally, the total cell counts in BALF are important indicators of the proliferation and differentiation of inflammatory cells. After infection of wild-type mice, total cell counts in the *S. mitis* group were hardly increased, whereas those in the PAO1 group were significantly increased compared with the PBS group. These findings are consistent with the results of lung histopathological examination. However, the total cell counts in the PAO1+ *S. mitis* group were slightly lower than those in the PAO1 group. We speculate that *S. mitis* might activate some other signaling pathways in the process of promoting inflammatory cells. Some studies have found that *S. mitis* reduces proliferation of T cells specific to an unrelated antigen (TT), which suggests an inhibitory effect of *S. mitis*. This inhibition can affect either the antigen-presenting cells (APCs) directly by preventing activation and/or antigen presentation or on the memory Th cells directly by interfering with the APC–T cell interaction [5]. We speculate that this might be one possible explanation for our observations. After infection of TLR4-deficient mice, the total cell counts were significantly lower than those in wild-type mice, which indicates that TLR4 expressed on cell surface is needed for inflammatory cell activation [25].

Several studies have demonstrated that TNF-α activates the expression of endothelial adhesion molecules to facilitate migration of neutrophils into inflamed lungs [26]. Similarly, in our acute lung infection mouse models, we found that wild-type mice infected with PAO1 showed significantly increased TNF-α protein expression as well as increased IL-6 expression. These findings are consistent with the literature, which supportes that IL-6 modulates almost every aspect of the innate immune system, including the accumulation of neutrophils at sites of infection or trauma through the control of granulopoiesis [27, 28]. Previous studies have confirmed that TLR4 plays a crucial role in the recognition of Gram-negative bacteria, but has no obvious effects on Gram-positive bacteria [25, 29]. However, our present study showed that *P. aeruginosa* together with *S. mitis* could affect activity of the TLR4 pathway. Although we examined the TLR4 pathway specifically, the roles of other TLR pathways cannot be excluded and should be considered in future investigations.

In contrast with our study, Wang et al. found an opposite result, which confirmed that *S. mitis* mainly secrets autoinducer-2 to promote biofilm formation and to increase proinflammatory cytokine production, resulting in severe inflammation [6]. This difference might be attributed to the different infection models. In their study, chronic pneumonia rat models were established by dual-biofilm catheter intubation for 7 days. In contrast, in our study, acute lung infection mouse models were established by planktonic bacteria infection for only 48 h. However, further investigations are needed to elaborate the mechanisms. Additionally, we speculate that the conflicting results are related to the protective effects of *S. mitis* in acute infection, which resulted in reduced cytokine secretion by the host immune system and weakened bacterial clearance, finally resulting in persistent and chronic infection.

## Conclusions

In conclusion, the results of this study will contribute to a better understanding of the co-existence of *P. aeruginosa* and *S. mitis*, and for the first time, we demonstrate that *S. mitis* plays a potentially protective role in the respiratory tract during acute infection*.* These findings may provide a new perspective in the treatment of *P. aeruginosa* infection in the NICU. The mechanisms underlying the protective effect of *S. mitis* remain unknown, and further studies are needed to discover whether this effect is mediated by TLR signaling directly or via some small molecules or whether *S. mitis* simply competes with PAO1 for nutrients, limiting PAO1 replication.

## Abbreviations

BALF: Bronchoalveolar lavage fluid
CFU: Colony-forming units
ETTs: Endotracheal tubes
H&E: Hematoxylin-eosin
LPS: Lipopolysaccharide
*P. aeruginosa*: *Pseudomonas aeruginosa*
PAMPs: Pathogen-associated molecular patterns
PBS: Phosphate-buffered saline
*S. mitis*: *Streptococcus mitis*
TLR: Toll-like receptor
TT: Unrelated antigen
VAP: Ventilator-associated pneumonia
